# Supplementary material for: Hybrid spin Hall nano-oscillators based on ferromagnetic metal/ferrimagnetic insulator heterostructures
Source: Nat Commun. 2023 Mar 14;14:1406. doi: 10.1038/s41467-023-37028-4 (PMC10015054; doi:10.1038/s41467-023-37028-4)
Supplement: Supplementary file 1 — Supplementary information [file 41467_2023_37028_MOESM1_ESM.pdf]

Supplementary materials: Hybrid spin Hall nano-oscillators based on ferromagnetic metal/ferrimagnetic insulator heterostructures

Haowen Ren[1], Xin Yu Zheng[2], Sanyum Channa[3], Guanzhong Wu[1], Daisy A. O'Mahoney[4], Yuri Suzuki[2], and Andrew D. Kent[1]

[1]Center for Quantum Phenomena, Department of Physics, New York University, New York, NY 10003, USA

[2]Department of Applied Physics and Geballe Laboratory for Advanced Materials, Stanford University, Stanford, CA 94305, USA

[3]Department of Physics and Geballe Laboratory for Advanced Materials, Stanford University, Stanford, CA 94305, USA

[4]Department of Materials Science and Engineering and Geballe Laboratory for Advanced Materials, Stanford University, Stanford, CA 94305, USA

Supplementary Note 1. Sample properties.

Magnetic properties determined by the FMR and ST-FMR measurements described in the main text are presented in Table S1. The resistance values of the nanowires and stripes are also reported. The Table S2 summarizes the devices properties at auto-oscillation state. Damping of LFO15 is not measurable with FMR due to its strong anisotropy and its value is set to be the same as LAFO20. The gyromagnetic ratio was fixed to $\gamma =$ 180 GHz/T during the fitting.

Supplementary Table S1. Parameters from FMR, ST-FMR and resistance measurements. As discussed in the main text, the FMR measurements were conducted on unpatterned thin films. ST-FMR measurements were on 2μm width wires and the resistance values are measurement on 2μm and 400nm nanowire samples.

Sample	Composition	FMR measurements						ST-FMR measurements (stripe sample)				Resistance	
		$\mu_0 M_{eff}$ (T)	$\mu_0 \overline{M}_{eff}$ (T)	H_a (T)	α	$\overline{\alpha}$	ΔH_0 (T)	$\mu_0 M_{eff}$ (T)	H_a (T)	α	I_{th} (mA)	NWs (Ω)	Stripes (Ω)
Py5/Pt5	Ni0.81Fe0.19	0.78	N/A	0.009	0.0228	N/A	14.1e-4	0.78	-1.1e-4	0.025	22	124	194
LAFO4	Li0.5Al1.0Fe1.5O4	0.71	N/A	-0.005	0.0027	N/A	7.5e-4	N/A	N/A	N/A	N/A	N/A	N/A
LAFO4/Py5/Pt5	Li0.5Al1.0Fe1.5O4	0.84	0.77	0.001	0.0151	0.0215	27.0e-4	0.76	6.5e-4	0.024	18.5	116	163
LAFO10	Li0.5Al1.0Fe1.5O4	0.94	N/A	-0.017	0.0013	N/A	4.7e-4	N/A	N/A	N/A	N/A	N/A	N/A
LAFO10/Py5/Pt5	Li0.5Al1.0Fe1.5O4	0.89	0.80	0	0.0151	0.0196	27.7e-4	0.81	-4.8e-4	0.025	24	190	184
LAFO20	Li0.5Al1.0Fe1.5O4	1.03	N/A	-0.022	0.0011	N/A	3.3e-4	N/A	N/A	N/A	N/A	N/A	N/A
LAFO20/Py5/Pt5	Li0.5Al1.0Fe1.5O4	0.95	0.84	-0.008	0.0081	0.0171	35.8e-4	0.89	-29e-4	0.018	26.5	141	182
LFO15	Li0.5Al0.5Fe2O4	1.85	N/A	-0.053	N/A	N/A	N/A	N/A	N/A	N/A	N/A	N/A	N/A
LFO15/Py5/Pt5	Li0.5Al0.5Fe2O4	1.25	1.14	-0.022	0.0066	0.0153	40.7e-4	0.99	43.4e-4	0.064	28	194	191

Supplementary Table S2. Summary of properties of 400 nm nanowire devices with different heterostructures in the auto-oscillation state obtained from ST-FMR and PSD maps.

Sample	Threshold current from NWs' ST-FMR I_{th} (mA)	Max auto-oscillation current I_{max} (mA)	Max signal over noise floor (dB)	Max Q factor
Py5/Pt5	4.7	3.7	1.39	110
LAFO4/Py5/Pt5	2.6	2.4	2.04	220
LAFO10/Py5/Pt5	2.4	3.0	7.48	350
LAFO20/Py5/Pt5	3.6	3.1	32.08	505
LFO15/Py5/Pt5	3.0	2.8	29.89	821

Supplementary Note 2. Anisotropic magnetoresistance.

The anisotropic magnetoresistance (AMR) of 2µm width wires was measured by rotating a saturating field in the sample plane. The measured AMR ratios in different samples are almost the same, showing that the change of output power is not caused by the change of AMR.

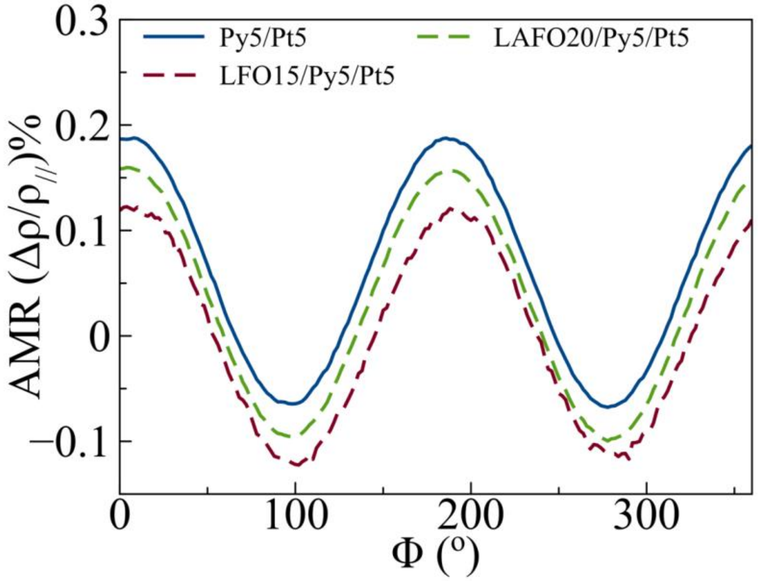

Supplementary Figure S1. AMR measurements conducted on Py5/Pt5, LAFO20/Py5/Pt5, and LFO15/Py5/Pt5 samples. The samples are patterned into 2-micron wide bars with a 3:1 aspect ratio. The external field was fixed at 0.2 T and samples are rotated in plane. ϕ=0° corresponds to the current parallel to the magnetic field.

Supplementary Note 3. Spin-torque FMR results.

ST-FMR measurements are carried out on both 2 μm stripe samples and 400 nm NW samples with different compositions. The magnetic field are applied at ϕ=70°. During the current bias sweeping scan, we fixed the RF frequency at 7 GHz.

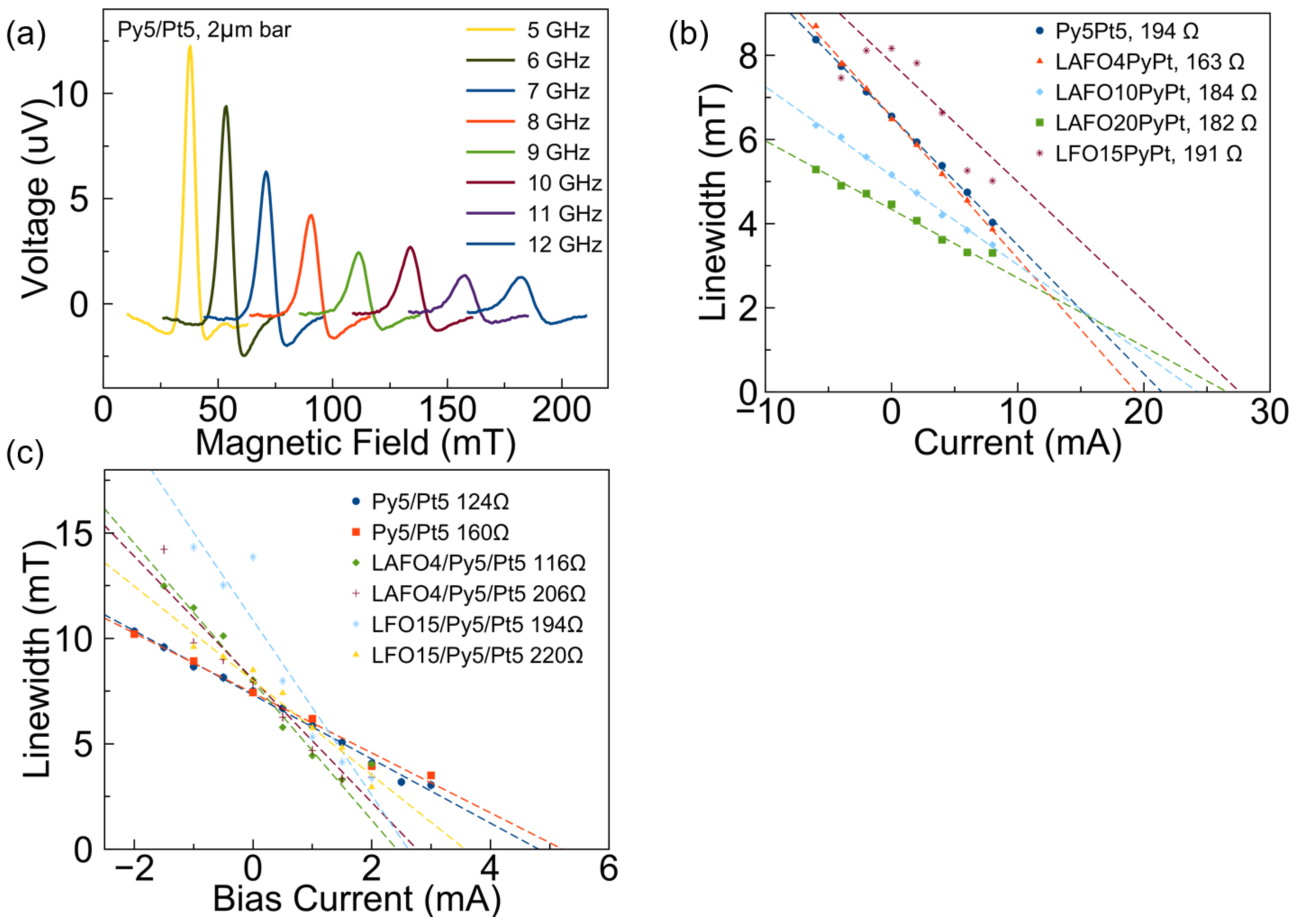

Supplementary Figure S2. (a) ST-FMR spectra of a 2μm wide Py5/Pt5 sample as a function of frequency. (b) Linewidth as a function of DC bias current obtained from ST-FMR measurements for different samples at 7 GHz frequency. (c) Linewidth as function of DC bias current for 400nm wide samples at 7 GHz frequency. The dashed lines extrapolate the fitted curves to their intersection with x-axis, which indicates the expected threshold current for auto-oscillations. The threshold currents are listed in Table S1.

Supplementary Note 4. Micromagnetic modeling.

In addition to the discussion of the micromagnetic simulation described in the Method section, here we provide more details regarding the parameters that we used in the simulations. The exchange stiffness A_{ex} of Py and LAFO are set to 13e-12 J/m and 4e-13 J/m, respectively. The interlayer exchange coupling strength between Py and LAFO are set by scaling the exchange coupling factor to half of the harmonic mean of two layers. $\mu_0 M_s$ of Py and LAFO are obtained from commonly used value from references and are set to 1.08 T and 0.0942 T, respectively. Since the FMR is measuring effective magnetization $\mu_0 M_{eff}$ and we know that $\mu_0 M_{eff} = \mu_0 M_s -$

$2K_u/M_s$, where K_u is the perpendicular anisotropy, we can mimic $\mu_0 M_{eff}$ by adding a perpendicular uniaxial anisotropy K_u in the simulation. Here, the K_u of Py and LAFO are setting to 1.29e6 J/m^3 and -0.32e6 J/m^3, respectively. For the spin current, the spin current polarization from spin-Hall effect is fixed to (0,1,0) direction. The simulation threshold currents density J_{th} for different samples are found to be 1.38e12, 1.46e12, 1.56e12, 1.68e12 A/m^2, respectively. To achieve maximum auto-oscillation state, simulation current density are set to be $J_{max} = 1.2\, J_{th}$, make it consistent with experimental observations. Table S3 summarized important parameters for the simulations. The Spin Hall angle for all simulations is set to 0.15.

Supplementary Table S3. Simulation parameters for different samples.

**Sample**	**Py $\mu_0 M_s$ (T)**	**LAFO $\mu_0 M_s$ (T)**	**Py $\mu_0 M_{eff}$ (T)**	**LAFO $\mu_0 M_{eff}$ (T)**	**Exchange coupling scale**	**Py damping**	**LAFO damping**	**Sim_J_{th} (e12 A/m^2)**	**Sim_J_{max} (e12 A/m^2)**
Py5/Pt5	1.08	-	0.78	-	-	0.0260	-	1.38	1.66
LAFO4/Py5/Pt5	1.08	0.0942	0.78	0.70	0.5	0.0260	0.0026	1.46	1.75
LAFO10/Py5/Pt5	1.08	0.0942	0.78	0.94	0.5	0.0260	0.0010	1.56	1.87
LAFO20/Py5/Pt5	1.08	0.0942	0.78	1.03	0.5	0.0260	0.0010	1.68	2.02

Micromagnetic simulations for Py5/Pt5 and LAFO20/Py5/Pt5 samples in an equilibrium state without applying any spin current are carried out. The transverse magnetization profiles are plotted in Fig. S3. From the transverse M_x profile (Fig. S3a), the magnetization at the edges of the LAFO20/Py5/Pt5 sample varies less than that of the Py5/Pt5 sample. Apart from that, the transverse M_z profile (Fig. S3b&c) clearly shows a much larger M_z component in LAFO20/Py5/Pt5 sample. Both provide evidence that by combining Py with LAFO, the edge mode will be more prominent compared to sample with a single Py layer.

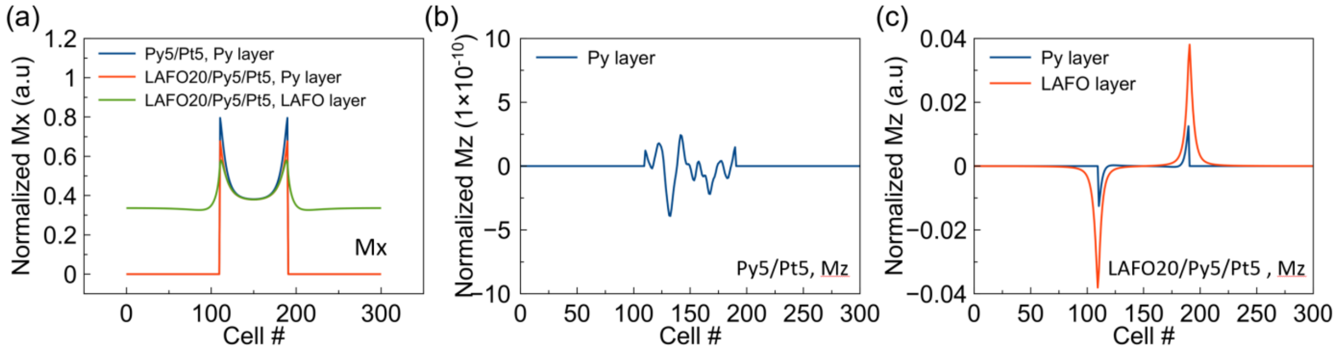

Supplementary Figure S3. (a) Transverse M_x profile of Py5/Pt5 and LAFO20/Py5/Pt5 samples. (b) Transverse M_z profile of Py5/Pt5 and (c) LAFO20/Py5/Pt5 samples.

Supplementary Note 5. Resonance field as a function of bias current obtained from ST-FMR.

One of the reasons for the redshift observed in PSD map in the Py5/Pt5 sample is the Joule heating caused by the current. However, as shown above in the resonance field as a function of bias current curves, the parabolic

curve is highly asymmetric about zero current, which indicates that the Joule heating is not the only contribution to the redshift in the PSD maps.

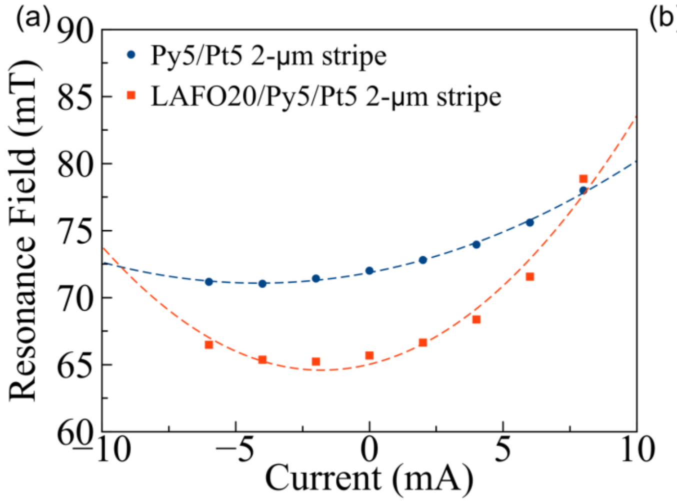

Supplementary Figure S4. Resonance field as a function of bias current for (a) 2µm stripe device and (b) 400-nm nanowire devices. Frequency is fixed at 7 GHz. Data are fitted with a parabolic function. Device made from Py5/Pt5 (LAFO20/Py5/Pt5) is labeled as blue circles (red squares).

Supplementary Note 6. Sample-to-sample variations

We show below a series of resistance, ST-FMR, and PSD measurements of additional devices. First of all, we measured resistances on different devices as shown in Table S4. We noticed that there is variation in the resistance within the devices that have the same geometry, especially the NW samples. This is mainly because of variations in the contact resistance in these two-terminal devices. Thus, as shown in Fig. S5, the resistance variations due to this contact resistance has negligible impact on the ST-FMR results from devices of the same geometry and composition. Finally, the PSD maps as a function of bias current for the additional samples are measured as shown in Fig. S6. The I_{max} obtained from the additional samples and their trend is consistent with previous samples. Thus, sample-to-sample variation is not affecting our conclusions.

Supplementary Table S4. Resistance measurements on nanowire and stripe samples.

Sample	Resistance NWs (Ω)	Resistance stripes (Ω)
Py5/Pt5	124&160	194&203
LAFO4/Py5/Pt5	116&206	163&178
LAFO15/Py5/Pt5	194&220	191&196

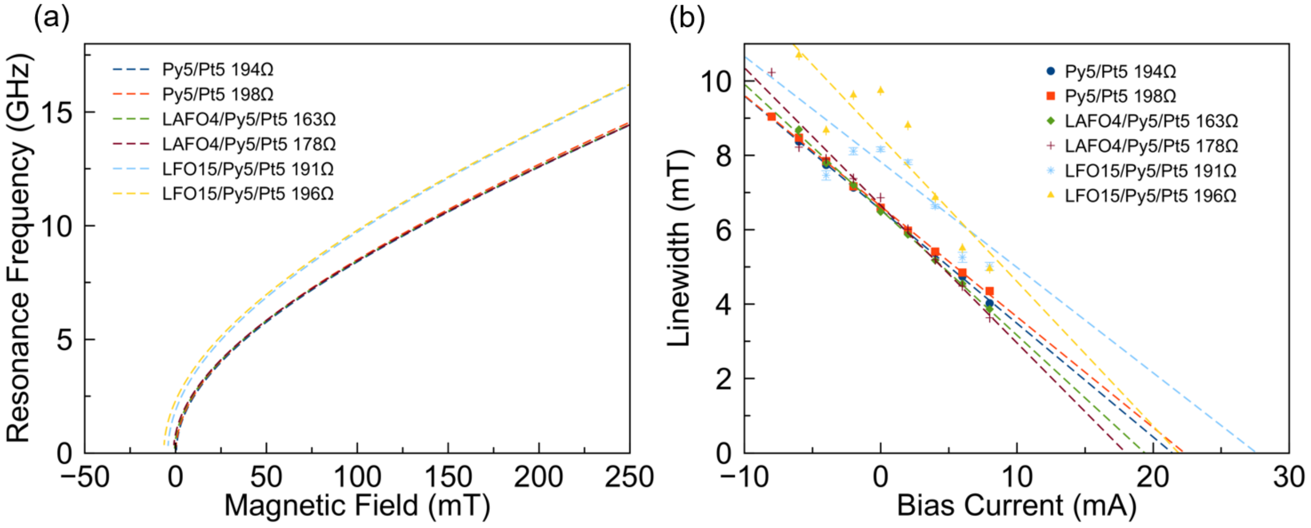

Supplementary Figure S5. Additional ST-FMR measurements on 2 μm stripe samples with two devices from each geometry and composition: (a) dispersion curves and (b) linewidth as a function of bias current.

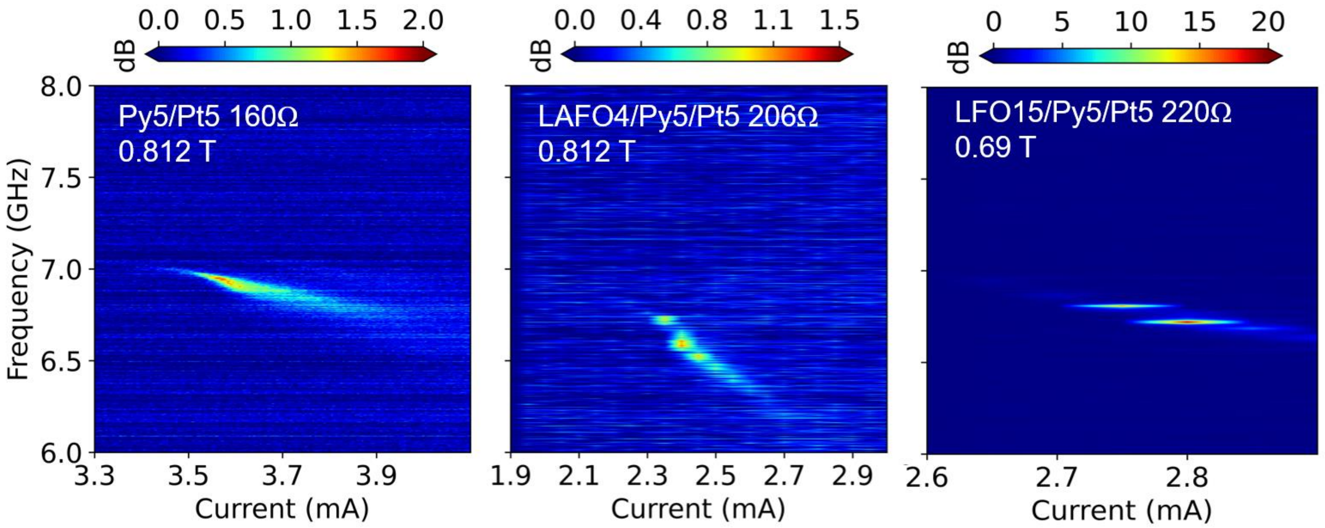

Supplementary Figure S6. Additional PSD maps on 400 nm nanowires samples as listed in Table S4. The results are consistent with previous reported results.

Supplementary Note 7. Ferromagnetic resonance of two strongly coupled ferromagnetic layers

This note develop and analyze a model for the ferromagnetic resonance response of two strongly ferromagnetically coupled magnetic layers.

1). Landau-Lifshitz-Gilbert equations: The starting point is the Landau-Lifshitz-Gilbert (LLG) equations for the magnetic moments of the ferromagnetic metal (layer 1: Permalloy, Py) and the ferrimagnetic insulating layer (layer 2: lithium aluminum ferrite (LAFO)). $\vec{m}_1 = M_{s,1}V_1\hat{m}_1$ and $\vec{m}_2 = M_{s,2}V_2\hat{m}_2$. Here $M_{s,i}$ is the magnetization,

V_i is the volume and $\hat{m}_i$ is a unit vector in the direction of the magnetic moments of the ith layer. The LLG equations are then

$$\frac{d\vec{m}_1}{dt} = \gamma_1 \vec{m}_1 \times \vec{H}_{\text{eff},1} \ - \frac{\alpha_1}{m_1} \vec{m}_1 \times \frac{d\vec{m}_1}{dt}, \tag{1}$$

$$\frac{d\vec{m}_2}{dt} = \gamma \vec{m}_2 \times \vec{H}_{\text{eff},2} - \frac{\alpha_2}{m_2} \vec{m}_2 \times \frac{d\vec{m}_2}{dt}, \tag{2}$$

where γ is the gyromagnetic ratio, α is the damping constant and H_{eff} is the effective field:

$$\vec{H}_{\text{eff},i} = -\frac{1}{m_i} \frac{\partial E}{\partial \hat{m}_i}, \tag{3}$$

The magnetic energy is given by:

$$E = -J \vec{m}_1 \cdot \vec{m}_2 - (\vec{m}_1 + \vec{m}_2) \cdot \vec{H} + \frac{1}{2} H_{A,1} m_1 (\hat{m}_1 \cdot \hat{z})^2 + \frac{1}{2} H_{A,2} m_2 (\hat{m}_2 \cdot \hat{z})^2, \tag{4}$$

where the $H_{A,i}$ characterizes the magnetic anisotropy; for $H_{A,i} > 0$ there is an xy-easy-plane magnetic anisotropy. The coupled LLG equations thus read:

$$\frac{\partial \vec{m}_1}{dt} = \gamma_1 \vec{m}_1 \times \left(J \vec{m}_2 + \vec{H} - H_{A,1} (\hat{m}_1 \cdot \hat{z}) \hat{z}\right) - \frac{\alpha_1}{m_1} \vec{m}_1 \times \frac{d\vec{m}_1}{dt}, \tag{5}$$

$$\frac{\partial \vec{m}_2}{dt} = \gamma_2 \vec{m}_2 \times \left(J \vec{m}_1 + \vec{H} - H_{A,2} (\hat{m}_2 \cdot \hat{z}) \hat{z}\right) - \frac{\alpha_2}{m_2} \vec{m}_2 \times \frac{d\vec{m}_2}{dt}. \tag{6}$$

In order to characterize the coupled modes we change variables to:

$$\hat{m} = \frac{1}{2} (\hat{m}_1 + \hat{m}_2), \tag{7}$$

$$\hat{m}_{\text{D}} = \frac{1}{2} (\hat{m}_1 - \hat{m}_2). \tag{8}$$

The resulting equation for the acoustic mode of the coupled layers net moment $\vec{m} = \vec{m}_1 + \vec{m}_2$ (i.e., the mode with $\hat{m}_D = 0$, taking $\gamma \equiv \gamma_1 = \gamma_2$) is:

$$\frac{d\vec{m}}{dt} = \gamma \vec{m} \times \left(\vec{H} - H_A \hat{z}\right) - \frac{1}{m} \left(\frac{\alpha_1 m_1 + \alpha_2 m_2}{m_1 + m_2}\right) \vec{m} \times \frac{d\vec{m}}{dt}, \tag{9}$$

where H_A is a linear combination of the magnetic anisotropies of the layers, i.e.:

$$H_A = \frac{H_{A,1} m_1 + H_{A,2} m_2}{m_1 + m_2} \tag{10}$$

Writing Eq. 9 as

$$\frac{d\vec{m}}{dt} = \gamma \vec{m} \times \left(\vec{H} - H_A \hat{z}\right) - \frac{\alpha_{\text{eff}}}{m} \vec{m} \times \frac{d\vec{m}}{dt}, \tag{11}$$

we see that the effective damping is a weighted sum of the damping of the two layers:

$$\alpha_{\text{eff}} = \frac{\alpha_1 m_1 + \alpha_2 m_2}{m_1 + m_2} \tag{12}$$

In the main text we write the effective anisotropy H_A using the symbol $\overline{M}_{\text{eff}}$ ($\equiv H_A$) and in terms of the magnetization and thickness of the layers in our samples this is given by

$$\overline{M}_{\text{eff}} = \frac{M_{s,\text{Py}} t_{\text{Py}} M_{\text{eff,Py}} + M_{s,\text{LAFO}} t_{\text{LAFO}} M_{\text{eff,LAFO}}}{M_{s,\text{Py}} t_{\text{Py}} + M_{s,\text{LAFO}} t_{\text{LAFO}}} \tag{13}$$

And the effective damping as $\bar{\alpha}$ ($\equiv \alpha_{\text{eff}}$):

$$\bar{\alpha} = \frac{M_{s,\text{Py}} t_{\text{Py}} \alpha_{\text{Py}} + M_{s,\text{LAFO}} t_{\text{LAFO}} \alpha_{\text{LAFO}}}{M_{s,\text{Py}} t_{\text{Py}} + M_{s,\text{LAFO}} t_{\text{LAFO}}} \tag{14}$$

In the strongly coupled case the threshold current for spin-wave excitations is proportional to:

$$I_{th} \propto \alpha_{\text{eff}} (m_1 + m_2) \overline{M}_{\text{eff}} \tag{15}$$

and thus is proportional to the damping and thicknesses of the magnetic layers:

$$I_{th} \propto \left(\alpha_{Py} M_{s,\text{Py}} t_{\text{Py}} + \alpha_{LAFO} M_{s,\text{LAFO}} t_{\text{LAFO}}\right) \overline{M}_{\text{eff}} \tag{16}$$
